# Proteomic analysis of seminal plasma in Bali-polled bulls: Identifying biomarkers for fertility assessment

**DOI:** 10.5455/javar.2025.l933

**Published:** 2025-07-02

**Authors:** Athhar Manabi Diansyah, Santoso Santoso, Herdis Herdis, Hasbi Hasbi, Takdir Saili, Suyatno Suyatno, Syahruddin Said, Tatan Kostaman, Maman Surachman, Fitra Aji Pamungkas, Umi Adiati, Rahma Isartina Anwar, Hikmayani Iskandar, Rahmat Rahmat, Hasrin Hasrin

**Affiliations:** 1 Faculty of Animal Science, Hasanuddin University, Makassar, Indonesia; 2 Research Center for Animal Husbandry, National Research and Innovation Agency, Cibinong Science Center, Bogor, Indonesia; 3 Research Center for Applied Zoology, National Research and Innovation Agency, Bogor, Indonesia; 4 Faculty of Animal Husbandry, Halu Oleo University, Kendari, Indonesia; 5 Faculty of Agriculture, Lambung Mangkurat University, Banjarbaru, Indonesia; 6 Faculty of Vocation, Hasanuddin University, Makassar, Indonesia

**Keywords:** Bali-polled bulls, seminal plasma, protein, biomarker, fertility

## Abstract

**Objective:**

This study aimed to conduct a proteomic analysis of the seminal plasma using advanced proteomic techniques in Bali-polled bulls in order to assess fertility.

**Materials and Methods:**

Semen samples from Bali-polled bulls (*n =* 5) were analyzed via one-dimensional sodium dodecyl sulfate-polyacrylamide gel electrophoresis for protein separation, followed by liquid chromatography–mass spectrometry for peptide identification. Data were processed using the UniProt bovine protein database and STRING software.

**Result:**

A total of 84 proteins were identified, with two remaining unclassified. These proteins were categorized into key biological processes, including reproduction, regulation of biological quality, and catabolic processes. Molecular functions predominantly included catalytic activity, hydrolase activity, and peptidase regulator activity, while cellular components were primarily associated with the extracellular region, vesicles, and lysosomes. Thirteen proteins were identified as directly linked to fertility functions: SPADH1, Binder of Sperm Proteins 3, SPADH2, ANG, Binder of Sperm Proteins 5, TEX101, ELSPBP1, FOLR3, TCP1, LDHC, HEXA, SPAM1, and CTSB. These proteins were further analyzed for their functional relevance to reproductive biology.

**Conclusion:**

While these findings provide preliminary insights into fertility-related biomarkers for Bali-polled bulls, further validation and statistical analysis are required to confirm their diagnostic and breeding potential. This proteomic analysis represents a first step toward understanding the molecular mechanisms of fertility in this underrepresented breed, contributing to improved breeding strategies and genetic resource management. Future studies should aim to expand the sample size, validate the identified biomarkers through experimental assays, and explore their potential for broader applications in bovine fertility management.

## Introduction

Bali-polled bulls, an indigenous cattle breed of Indonesia, are highly valued for their adaptability to tropical climates, resistance to local diseases, and their critical role in supporting smallholder farming systems [1]. However, their suboptimal fertility remains a significant challenge for sustainable livestock production. Despite these strengths, suboptimal reproductive performance poses a major obstacle to sustainable breeding and conservation programs. Low fertility rates hinder genetic improvement efforts and limit the breed’s potential to meet the growing demands of livestock production [2]. Low fertility rates hinder genetic improvement efforts and compromise the breed‘s potential to support the growing demands of livestock production [3]. These challenges are further compounded by a lack of understanding of the biological and molecular factors influencing fertility in Bali-polled bulls, leaving breeders without targeted tools to manage reproductive efficiency [4].

While fertility biomarkers in well-studied breeds such as *Bos taurus* and *Bos indicus* have been extensively explored [3], these findings do not directly apply to Bali-polled bulls due to their distinct genetic traits and environmental adaptations, which may affect fertility mechanisms differently [5]. Proteomic studies in *B. taurus* have highlighted the critical roles of seminal plasma proteins in sperm protection, capacitation, and fertilization [6], while research on *B. indicus* has shown how proteomic profiles influence fertility under stress conditions [7]. A significant gap exists in our understanding of the proteomic profiles of indigenous breeds, including Bali-polled bulls, which hinders the development of breed-specific fertility biomarkers that could address their unique reproductive challenges [8].

This study aims to characterize the seminal plasma proteome of Bali-polled bulls using advanced proteomic techniques to identify proteins that may be associated with key fertility functions, such as sperm capacitation, motility, and fertilization. These associations will be explored in relation to the biochemical properties of the semen, rather than directly measuring fertility outcomes. By exploring the interaction networks of these proteins using bioinformatic tools, the research aims to predict their potential roles in fertility-related processes, such as sperm function and fertilization, based on available literature and molecular data.

Additionally, by comparing the seminal plasma proteome of Bali-polled bulls with those of *B. taurus* and *B. indicus*, this study seeks to uncover both conserved and unique features, which may inform our understanding of breed-specific fertility traits. The insights gained will deepen our understanding of the molecular mechanisms behind fertility-related processes in Bali-polled bulls and contribute to the development of targeted strategies to enhance fertility management and genetic conservation for this indigenous breed. By identifying fertility-associated proteins in Bali-polled bulls and comparing their proteomic profiles with those of other breeds, this research will contribute to a broader understanding of reproductive biology and support sustainable breeding strategies for this breed.

## Materials and Methods

### Animals and ethical clearance

Frozen semen was obtained from Bali-polled bulls (*n =* 5), aged 9–10 years, with six batch codes, each consisting of five straws. The bulls with an average body weight of 300–500 kg were maintained according to the standard ethical protocol of animal care by the Artificial Insemination Center as the superior bulls. The Animal Ethics Commission of Hasanuddin University approved this study’s animal models and experimental designs with certificate number 302/UN4.6.4.5.31/pp36/2021. The production of frozen semen adhered to the Indonesian National Standard (SNI 4869-1: 2021) [9], ensuring the standardization of collection, processing, and preservation procedures in line with national guidelines.

### Semen samples

The semen samples were collected from Bali-polled bulls (*n =* 5) using artificial vaginas twice a week throughout the experiment and transferred into the Semen Processing Laboratory [10]. The seminal plasma was separated by centrifugation at 1,800 rpm for 30 min at 4°C, and the resulting supernatant was stored at −20°C for further analysis. Proteins were extracted using a buffer solution composed of 62.5 mM Tris-HCl (pH 6.8), 2% sodium dodecyl sulfate (SDS), 1.0 mM phenylmethanesulfonyl fluoride, and 23 mM benzidine as a protease inhibitor [11].

### Proteomic quality control and standards

All proteomic analyses were performed following the quality control measures recommended by the Minimum Information About a Proteomics Experiment guidelines. This included replicate analyses, blank controls, and validation of peptide identifications through multiple search engines to confirm protein reliability. This approach ensures the accuracy and reproducibility of the data obtained and complies with internationally recognized standards for proteomic experiments.

### SDS-polyacrylamide gel electrophoresis and staining gel

Protein characterization was performed using one-dimensional SDS-polyacrylamide gel electrophoresis to separate proteins by molecular weight [12]. Each sample (20 µg of seminal plasma protein) was resolved on a 12% polyacrylamide gel, using a PM2700 Excel-Band 3-color Broad Range marker (SMOBIO, Hsinchu, Taiwan) to identify molecular weights ranging from 5 to 245 kDa. Electrophoresis was carried out at 120 V for 70 min. After electrophoresis, the gels were stained with Coomassie Brilliant Blue R-250 and destained to allow visualization of protein bands. The bands were analyzed based on molecular weight and retention factor using Thermo SkanIt RE Multiskan Go software (version 3.2; Thermo Fisher Scientific, Waltham, MA), and band intensities were quantified with ImageJ software for precise data interpretation [13].

The visible protein bands were carefully excised from the gel, and further digestion protocols were followed for downstream analysis [14]. The gel bands were digested with trypsin (or an alternative enzyme) for peptide generation prior to liquid chromatography–mass spectrometry (LC-MS/MS) analysis. The digestion protocol involved incubation with sequencing-grade trypsin at a 1:50 enzyme-to-protein ratio at 37°C for 16 h, followed by peptide extraction and purification steps [8].

### Protein quantification

Seminal plasma proteins (25 µg per sample) were aliquoted into microtubes and vacuum-dried. Samples were incubated with 0.02 M TEAB, 5 mM DTT, and 1 mM CaCl₂ at 55°C for 25 min under agitation (400 rpm) in an Eppendorf^®^ Thermomixer^®^ R (Sigma-Aldrich, Darmstadt, Germany). Digested peptides were purified using C18 Spin Columns (Thermo Scientific, Pierce Biotechnology, Rockford, IL) packed with porous C18 reverse-phase resin. Peptides were eluted with 20 µl of 70% acetonitrile elution buffer and stored at −80°C for LC-MS/MS analysis [15].

### LC-MS/MS

Peptides were analyzed using LC-MS/MS on an Ultimate 3000 Nano LC system, coupled with a Q-Exactive Plus Orbitrap high-resolution mass spectrometer (Thermo Fisher Scientific, Bremen, Germany), following the protocols adapted from Diansyah et al. [4]. The peptide signals were acquired using an LTQ-Orbitrap mass spectrometer, with MS1 survey scans performed in the 200–2000 m/z range at a resolution of 30,000 (at m/z 400) [16]. A targeted MS2 approach was utilized for high-confidence peptide identification, with dynamic exclusion settings to ensure comprehensive sampling of peptides across the analyzed samples.

### Protein identification

Proteins were identified using Proteome Discoverer software (version 2.2, Thermo Fisher Scientific), searching against the UniProt bovine protein database. The search criteria included a precursor mass tolerance of 10 ppm, a fragment mass tolerance of 0.02 Da, and a minimum of two unique peptides per protein. Gene ontology annotations categorize proteins by biological functions, molecular functions, and cellular components. STRING database (http://string-db.org) was used for pathway and interaction network analysis. It is acknowledged that the use of a *B. taurus*-based protein database may not capture Bali-polled breed-specific proteins. This limitation may lead to the missed identification of proteins that are unique to the Bali-polled breed. Future studies will aim to overcome this hurdle by incorporating breed-specific genomic and proteomic resources to enhance the accuracy and sensitivity of protein identification in Bali-polled bulls.

## Results

### Protein identification

The seminal plasma proteome of Bali-polled bulls was characterized using data primarily derived from the *B. taurus* database, due to the limited availability of *Bos javanicus*-specific protein data. While *B. taurus* is commonly used as a reference for bovine proteomics, we acknowledge that the Bali-polled breed may have specific proteomic characteristics not captured by this database. A total of 84 proteins were identified using UniProt, with two remaining unclassified (Table 1). Of these, 13 proteins were associated with fertility functions, offering significant insights into the reproductive biology of Bali-polled bulls. A comparative analysis of these proteins with those from other cattle breeds with higher fertility rates would offer a more nuanced understanding of fertility in Bali-polled bulls. These fertility-associated proteins include SPADH1, Binder of Sperm Proteins 3 (BSP3), SPADH2, ANG, Binder of Sperm Proteins 5 (BSP5), TEX101, ELSPBP1, FOLR3, TCP1, LDHC, HEXA, SPAM1, and CTSB. Notably, SPADH1 emerged as a central node interacting with several other proteins, suggesting its critical role in fertility mechanisms (Fig. 4a). Further investigation is needed to quantify the expression of SPADH1 across individual bulls and evaluate its contribution to fertility in Bali-polled bulls specifically.

**Table 1.** The seminal plasma proteins of Bali-polled bulls.

**Gene symbol**	**Accession number**	**Description**	**Unique peptides**	**MW (kDa)**	**Molecular function**
SPADH2	P82292	Spermadhesin Z13	2	13.4	Single fertilization
GPX5	F1MCF5	Glutathione peroxidase	2	15.6	Peroxidase activity
SFP3	P04557	Seminal plasma protein A3	2	16.1	Sperm capacitation
WFDC2	G3MX65	WAP four-disulfide core domain protein 2	2	17.6	Aspartic-type endopeptidase inhibitor activity
AK1	P00570	Adenylate kinase isoenzyme 1	2	21.7	Kinase activity
RAB1A	A1L528	Ras-related protein Rab-1A	2	22.7	GTP binding
RAB14	Q3ZBG1	Ras-related protein Rab-14	2	23.9	GTP binding
FAM3C	A5PKI3	Protein FAM3C	2	24.8	Carbohydrate binding
PEB4P	Q3T010	Phosphatidylethanolamine-binding protein 4	2	25.1	Structural constituent of ribosome
ABHD14	E1BDS4	Abhydrolase domain containing 14A	2	25.8	Hydrolase activity
CD81	Q3ZCD0	CD81 antigen	2	25.8	Cholesterol binding
TFPI2	Q7YRQ8	Tissue factor pathway inhibitor 2	2	26.7	Serine-type endopeptidase inhibitor activity
NGF	P13600	Beta-nerve growth factor	2	26.7	Cholesterol binding
CLIC1	Q5E9B7	Chloride intracellular channel protein 1	2	27	Oxidoreductase activity
TEX101	A6QPE3	TEX101 protein	2	27.3	Regulation of flagellated sperm motility
ESTD	Q08E20	S-formylglutathione hydrolase	2	31.5	S-formylglutathione hydrolase activity
RNASET2	Q0III8	RNASET2 protein	2	32.8	RNA binding
LOC507756	E1BI74	NAD(P)(+)--arginine ADP-ribosyltransferase	2	33.7	NAD+-protein-arginine ADP-ribosyltransferase activity
ZA2G	Q3ZCH5	Zinc-alpha-2-glycoprotein	2	33.8	Immune response
PRSS22	E1BNJ9	Serine protease 22	2	34.1	Serine-type endopeptidase activity
PPT1	F1MSA1	Palmitoyl-protein thioesterase 1	2	34.1	Palmitoyl-(protein) hydrolase activity
PTGR1	F1N2W0	Prostaglandin reductase 1	2	35.7	15-oxoprostaglandin 13-oxidase activity
N/A	G3N0V0	Ig-like domain-containing protein	2	35.9	Immunoglobulin receptor binding
PRSS8	F1MUB9	Serine protease 8	2	36.7	Positive regulation of sodium ion transport
N/A	B2KJ42	Fructose-1,6-bisphosphatase 1	2	36.7	Fructose 1,6-bisphosphate 1-phosphatase activity
FUCA2	F1N5H2	Alpha-L-fucosidase	2	53.6	Alpha-L-fucosidase activity
ALDH9A1	F1N2L9	4-trimethylaminobutyraldehyde dehydrogenase	2	54	4-trimethylammoniobutyraldehyde dehydrogenase activity
SERPINF2	P28800	Alpha-2-antiplasmin	2	54.7	Serine-type endopeptidase inhibitor activity
NUCB1	Q0P569	Nucleobindin-1	2	54.9	DNA binding
APCDD1	F1MNJ4	Protein APCDD1	2	55.9	Protein binding
BTD	A6QQ07	Biotinidase	2	58.3	Biotinides activity
TCP1	G5E531	T-complex protein 1 subunit alpha	2	60.2	ATP binding
EHD1	Q5E9R3	EH domain-containing protein 1	2	60.6	ATP binding
PKM2	Q3ZC87	Pyruvate kinase	2	61.4	ATP binding
IDS	F1N2D5	Iduronate 2-sulfatase	2	61.4	Iduronate-2-sulfatase activity
QSOX1	A6QQA8	Sulfhydryl oxidase	2	62.9	Flavin-dependent sulfhydryl oxidase activity
KRT1	G3N0V2	Keratin, type II cytoskeletal 1	2	63.1	Carbohydrate binding
EZR	P31976	Ezrin	2	68.7	Cell adhesion molecule binding
SMPD1	Q0VD19	Sphingomyelin phosphodiesterase	2	69.3	Zinc ion binding
RNASE4	Q58DP6	Ribonuclease 4	3	16.9	RNA activity
ANG	A6H6X2	ANG protein	3	17.1	RNA activity
N/A	F1N1Z8	Uncharacterized protein	3	22	-
RAB2B	E1BC58	RAB2B, member RAS onco family	3	24.2	GTP binding
TIMP2	F1N430	Metalloproteinase inhibitor 2	3	24.4	Protease binding
N/A	G3N2N9	Uncharacterized protein	3	25.1	-
GPX3	P37141	Glutathione peroxidase 3	3	25.6	Selenium binding
FAM3B	E1BQ21	FAM3 metabolism-regulating signaling molecule B	3	26	Carbohydrate binding
PLET1	A5D7U1	Placenta-expressed transcript 1 protein	3	26.6	Regulation of cell migration
CRISP3	Q3ZCL0	Cysteine-rich secretory protein 2	3	27.4	-
IFI30	A6QPN6	Gamma-interferon-inducible lysosomal thiol reductase	3	27.5	Oxidoreductase activity, acting on a sulfur group of donors
FOLR3	P02702	Folate receptor alpha	3	27.9	Signaling receptor activity
STX12	A7MAZ2	STX12 protein	3	31.3	SNAP receptor activity
MDH1	Q3T145	Malate dehydrogenase, cytoplasmic	3	36.4	Hydroxyphenylpyruvate reductase activity
C1GALT1C1	Q3SX46	C1GALT1-specific chaperone 1	3	36.4	Glycoprotein-N-acetylgalactosamine 3-beta-galactosyltransferase activity
LOC101907989	G3MX67	Protein LEG1 homolog	3	36.5	-
SERPINC1	F1MSZ6	Antithrombin-III	3	52.4	Protease binding
BPI	P17453	Bactericidal permeability-increasing protein	3	53.4	Lipopolysaccharide binding
P4HB	A6H7J6	Protein disulfide-isomerase	3	57.2	Disulfide isomerase activity
SIAE	Q2KI90	Sialic acid acetylesterase	3	59.7	Sialate O-acetylesterase activity
TPP1	F1MK08	Tripeptidyl peptidase 1	3	61.3	Serine-type endopeptidase activit
NT5E	Q05927	5’-nucleotidase	3	62.9	Protein binding
SLC3A2	Q08DL0	SLC3A2 protein	3	63.1	Protein binding
ALB	A0A140T897	Albumin	3	69.3	Fatty acid binding
SPADH1	P29392	Spermadhesin-1	4	15	Single fertilization
Akr1b1	Q862S5	Aldo-keto reductase family 1 member B1	4	16.1	NADPH activity
BSP5	P81019	Seminal plasma protein BSP-30 kDa	4	21.3	Single fertilization
LCN2	E1B6Z6	Lipocalin 2	4	23	Protein binding
STX7	Q3ZBT5	Syntaxin-7	4	29.6	SNAP receptor activity
CTSB	P07688	Cathepsin B	4	36.6	Endopeptidase activity
FUCA1	Q2KIM0	Tissue alpha-L-fucosidase	4	54.1	Alpha-L-fucosidase activity
KRT10	A6QNZ7	Keratin, type I cytoskeletal 10	4	54.8	Heterodimerization activity
VNN1	Q58CQ9	Pantetheinase	4	56.9	Pantetheine hydrolase activity
SGSH	E1BFX4	N-sulfoglucosamine sulfohydrolase	4	57.2	N-sulfoglucosamine sulfohydrolase activity
HEXA	Q0V8R6	Beta-hexosaminidase subunit alpha	4	60.3	Beta-N-acetylhexosaminidase activity
HPSE	F1N1G1	Heparanase	4	61.1	Heparanase activity
YWHAZ	P63103	14-3-3 protein zeta/delta	5	27.7	Phosphoserine residue binding
PGAM2	F1N2F2	Phosphoglycerate mutase	5	28.7	Bisphosphoglycerate mutase activity
APOA1	P15497	Apolipoprotein A-I	5	30.3	Cholesterol transfer activity
HEXB	H7BWW2	Beta-hexosaminidase	5	61.2	Beta-N-acetylhexosaminidase activity
LGALS3BP	A7E3W2	Galectin-3-binding protein	5	62.1	-
CST6	Q5DPW9	Cystatin E/M	6	16.3	Cysteine-type endopeptidase inhibitor activity
PEBP1	P13696	Phosphatidylethanolamine-binding protein 1	6	21	ATP binding
ELSPBP1	E1B9P4	Epididymal sperm-binding protein 1	6	26	Single fertilization
MDH2	Q32LG3	Malate dehydrogenase, mitochondrial	7	35.6	Homodimerization activity
SPAM1	F1MTV1	Hyaluronidase	7	62.2	Hyalurononglucosaminidase activity
LDHC	E1BNS9	L-lactate dehydrogenase	8	36	Flagellated sperm motility

### Functional annotations

Functional annotations of the identified proteins were performed using STRING software, categorizing them based on biological processes, molecular functions, and cellular components. However, the lack of quantification of protein levels across animals limits our understanding of how these proteins vary between individuals within the Bali-polled breed.

### Biological processes

**Figure 1.**
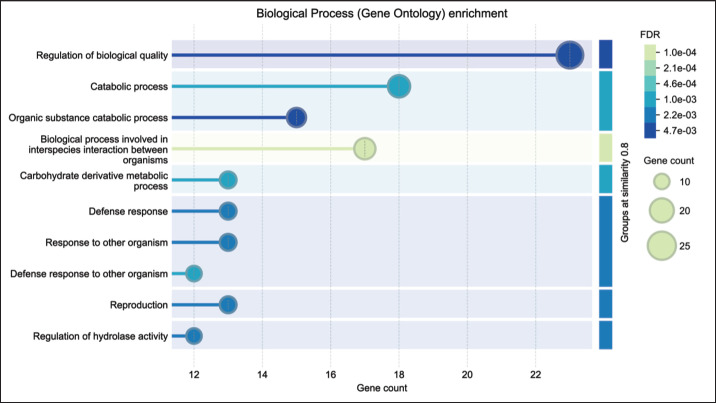
The biological process (gene ontology) enrichment of Bali-polled bulls.

Proteins were predominantly involved in the regulation of biological quality (23 proteins), followed by catabolic processes (18), organic substance catabolic processes (15), interspecies interactions (17), carbohydrate derivative metabolism (13), defense responses (13), reproduction (13), and regulation of hydrolase activity (12) (Fig. 1).

### Molecular functions

**Figure 2.**
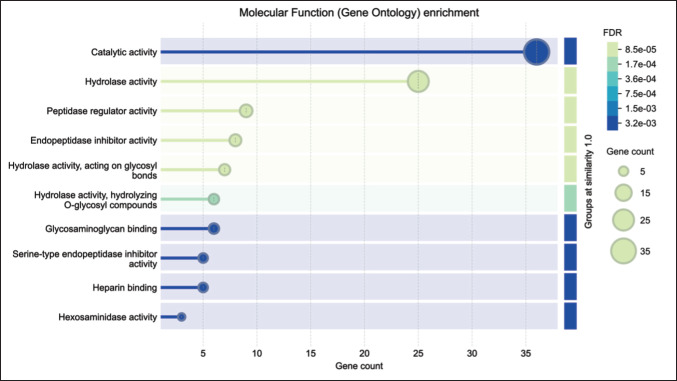
Molecular function (gene ontology) enrichment of Bali-polled bulls.

The predominant molecular functions included catalytic activity (36 proteins), hydrolase activity (25), peptidase regulator activity (9), and endopeptidase inhibitor activity (8). Other functions included hydrolase activity acting on glycosyl bonds (7), serine-type endopeptidase inhibitor activity (5), and heparin binding (5) (Fig. 2).

### Cellular components

**Figure 3.**
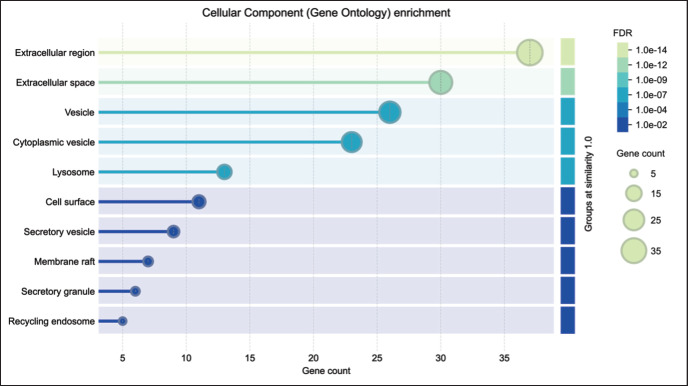
Cellular component (gene ontology) enrichment of Bali-polled bulls.

The proteins were primarily localized to the extracellular region (37 proteins) and extracellular space (33), with significant associations with vesicles (26), lysosomes (13), secretory granules (6), and recycling endosomes (5) (Fig. 3). Quantitative data on the expression levels of these proteins across individual bulls could enhance the interpretation of their functional roles in fertility.

**Figure 4.**
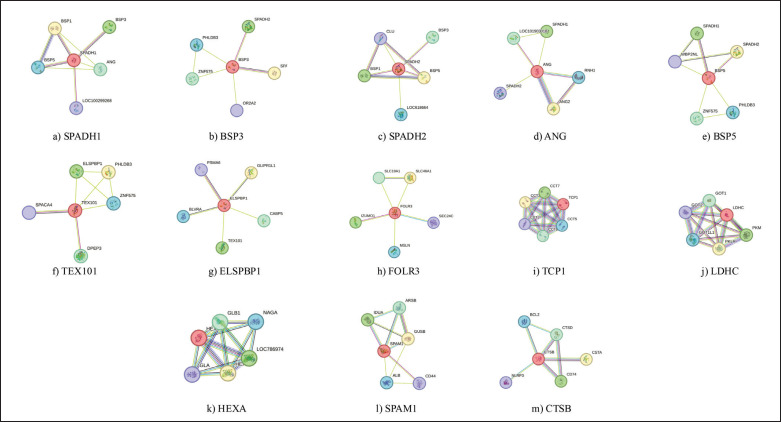
The proteins specific as biomarker candidate linked to fertility function in Bali-polled bulls: a) SPADH1, b) BSP3, c) SPADH2, d) ANG, e) BSP5, f) TEX101, g) ELSPBP1, h) FOLR3, i) TCP1, j) LDHC, k) HEXA, l) SPAM1, and m) CTSB.

### Protein interaction networks

STRING analysis provided detailed interaction networks for the 13 fertility-associated proteins, revealing key functional and structural relationships. However, it would be beneficial to include protein expression data to assess variability in protein interactions between individual Bali-polled bulls and compare them with those in other breeds. SPADH1 was a central node interacting with BSP3, BSP1, BSP5, ANG, and LOC100299268 (Fig. 4a), while BSP3 connected with SPADH1, SPADH2, PHLDB3, ZNF575, and OR2A2 (Fig. 4b). SPADH2 interacted with BSP1, BSP3, BSP5, CLU, and LOC618664 (Fig. 4c), and ANG was interconnected with SPADH1, SPADH2, ANG2, LOC101903018, and RNH1 (Fig. 4d). BSP5 was linked to SPADH1, SPADH2, WBP2NL, PHLDB3, and ZNF575 (Fig. 4e), while TEX101 was associated with SPACA4, ELSPBP1, PHLDB3, ZNF575, and DPEP3 (Fig. 4f). These networks highlight the interconnected roles of these proteins in fertility-related processes.

## Discussion

This study offers a detailed characterization of the seminal plasma proteome in Bali-polled bulls, identifying 13 proteins potentially linked to fertility (Table 1). This aligns with previous proteomic studies in other bovine breeds, which have similarly identified proteins involved in sperm function, capacitation, and fertilization [17]. However, a comparison of the fertility-associated proteins in Bali-polled bulls with those in breeds known for higher fertility could provide a clearer context for the implications of these findings. Notably, these proteins were involved in catalytic activity, molecular binding, and structural modifications essential for sperm functionality, consistent with roles observed in sperm biology across different species. While the enrichment of biological processes such as regulation of biological quality, reproduction, and oxidative stress responses in this study (Fig. 1) mirrors findings in other mammalian seminal fluids, the specific protein interactions in Bali-polled bulls require further validation. The identification of proteins such as SPADH1 and BSP5, involved in sperm capacitation and zona pellucida binding, is similar to their roles in other cattle breeds, reinforcing the relevance of these markers for fertility evaluation. However, the specific role of these proteins in Bali-polled bulls may differ and requires validation through direct experiments such as Western blot or enzyme-linked immunosorbent assay (ELISA). However, the role of these proteins in Bali-polled bulls could differ, and the variability in protein levels between individuals within this breed must be considered, warranting further investigation using a quantitative proteomic approach.

Among the identified proteins, SPADH1, SPADH2, BSP3, BSP5, TEX101, ELSPBP1, and ANG are known for their roles in sperm viability and fertilization readiness (Table 1). This corroborates previous research in bovine seminal plasma, where similar proteins were linked to capacitation and sperm-zona pellucida binding [18]. STRING analysis (Fig. 4a) revealed key protein interactions, such as SPADH1’s regulation of capacitation through its interaction with BSP proteins, aligning with observations in other breeds [19,20]. SPADH2, which stabilizes sperm membranes, complements these functions, contributing to sperm viability during transit, a finding consistent with studies on other cattle [17]. TEX101 and ELSPBP1 play crucial roles in sperm motility and the acrosome reaction (Fig. 4f and g), and their interdependence during fertilization has been described in other contexts as well [21]. While these proteins are likely involved in similar mechanisms in Bali-polled bulls, direct experimental validation in this breed is necessary to confirm their specific roles. BSP3 and BSP5 play crucial roles in capacitation and the acrosome reaction by remodeling the sperm membrane [22]. This finding is consistent with research in other bovine species, where BSP proteins have been shown to facilitate zona pellucida penetration through interaction with glycosaminoglycans and triggering tyrosine phosphorylation [23,24]. However, the specific contributions of BSP3 and BSP5 in Bali-polled bulls require further experimental confirmation to fully understand their mechanisms in this breed.

Proteins such as ANG and SPAM1, linked to sperm survival and extracellular matrix degradation, play critical roles in stress adaptation and egg penetration [25]. To assess their contribution to fertility in Bali-polled bulls, a more detailed quantitative approach is needed, such as assessing their levels in the seminal plasma of bulls with varying fertility rates. ANG’s protective role against oxidative stress (Fig. 4d) and SPAM1’s involvement in hyaluronic acid degradation for sperm passage through the cumulus oophorus (Fig. 4l) are consistent with findings in other bovine studies. These functions, although plausible and supported by prior literature, need experimental validation specific to Bali-polled bulls to fully assess their relevance in this breed. Although these functions are supported by prior literature [26,27], they remain speculative in the context of Bali-polled bulls without direct experimental validation. TCP1 and LDHC are central to energy metabolism and motility [28], with TCP1 stabilizing flagellar cytoskeletal structures for motility and LDHC providing Adenosine Triphosphate through glycolysis for sustained sperm movement (Fig. 4i and j). These roles align with findings from other bovine breeds, illustrating their involvement in metabolic pathways that support sperm function [29]. However, this conclusion is based on studies from other breeds, and direct measurements in Bali-polled bulls are needed to confirm their specific roles. EXA and CTSB are involved in capacitation and the acrosome reaction by remodeling glycoproteins and regulating proteolytic pathways, roles that align with those described in other cattle breeds [30]. STRING analysis (Fig. 4k and m) confirmed their involvement in enzymatic processes critical for zona pellucida penetration, ensuring sperm readiness for fertilization.

While this study provides valuable initial insights, several limitations must be acknowledged. The reliance on *B. taurus* databases for protein identification may overlook unique Bali-polled fertility markers. Additionally, the absence of a direct fertility trial or quantitative approach (such as protein expression profiling linked to fertility metrics) limits the ability to assess how the identified proteins correlate with actual fertility outcomes in Bali-polled bulls. Future studies should incorporate fertility trials to establish the functional roles of these proteins in fertility, as well as larger and more diverse cohorts of Bali-polled bulls to assess inter-animal variability in seminal protein expression.

Furthermore, the small sample size (five bulls) restricts the generalizability of the findings, and future studies should include a broader sample to strengthen the statistical power. The use of *B. taurus* databases may have excluded Bali-specific proteins, introducing bias and possibly overlooking unique fertility markers specific to Bali-polled bulls. Developing breed-specific genomic and proteomic databases should be a priority in future research. Finally, the lack of experimental validation (e.g., Western blot or ELISA) for key proteins limits the ability to draw firm conclusions about their functional roles in Bali-polled bull fertility, which is essential for validating the relevance of these proteins in the breed.

While this study will clarify the seminal plasma proteome and its potential relevance to fertility, claims about diagnostic applications and genetic conservation are premature. High levels of SPADH1, BSP5, TEX101, and ANG might suggest fertility potential, but such conclusions require further validation through functional assays and longitudinal studies, particularly in comparison to other breeds with higher fertility rates. Similarly, proteins such as LDHC and TCP1 could indicate metabolic fitness, while ELSPBP1 and FOLR3 might offer insights into genomic stability, but these roles must be confirmed in a Bali-polled bull-specific context. As a preliminary exploration, this study establishes a foundation for future work, but further research is needed to translate these findings into practical applications for fertility diagnostics and genetic conservation. Additionally, expanding the proteomic database to include Bali-polled-specific proteins will be essential for more accurate fertility assessments and conservation strategies.

## Conclusion

This study characterized the seminal plasma proteome of Bali-polled bulls. While these findings provide preliminary insights into fertility-related biomarkers for Bali-polled bulls, further validation and statistical analysis are required to confirm their diagnostic and breeding potential. This proteomic analysis represents a first step toward understanding the molecular mechanisms of fertility in this underrepresented breed, contributing to improved breeding strategies and genetic resource management.

## List of abbreviations

ELISA, enzyme-linked immunosorbent assay; h, hour; LC-MS/MS, liquid chromatography tandem−mass spectrometry; kg, kilogram; min, minutes; mM, millimolar; ppm, parts per million; SDS, sodium dodecyl sulfate; SNI, Indonesian National Standard; rpm, revolutions per minute; °C, degree Celsius; µg, microgram; µl, microliter.
